# Examining the Prevalence of Peripartum Depressive Symptoms in a Border Community

**DOI:** 10.1089/whr.2020.0105

**Published:** 2021-06-29

**Authors:** Iván A. de la Rosa, Junxin Huang, Charlotte C. Gard, Jill A. McDonald

**Affiliations:** ^1^ School of Social Work, Southwest Institute for Health Disparities Research, New Mexico State University, Las Cruces, New Mexico, USA.; ^2^ Department of Economics, Applied Statistics, and International Business and Southwest Institute for Health Disparities Research, New Mexico State University, Las Cruces, New Mexico, USA.; ^3^ Department of Public Health Sciences, Southwest Institute for Health Disparities Research, New Mexico State University, Las Cruces, New Mexico, USA.

**Keywords:** depression, peripartum period, Health Status Disparities, U.S.–Mexico border, Ethnic Groups

## Abstract

***Introduction:*** Depression is one of the most common complications in pregnancy, affecting 10% to 20% of women. Untreated peripartum depression increases the risk of adverse life events, more considerable distress, homelessness, and illness later in life. This study explored the prevalence of peripartum depression and associated demographic characteristics in a population of low-income, Healthy Start program participants in one New Mexico county along the U.S.–Mexico border where knowledge of depression prevalence is lacking.

***Materials and Methods:*** Healthy Start caseworkers routinely administered the 10-item Edinburgh Postnatal Depression Scale (EPDS) to all pregnant and recently pregnant program participants between 2009 and 2017. Scores for the first prenatal screen, first postpartum screen, and all screens for 1453 women were studied. A score of >10 points out of a possible 30 indicated a positive screen. Screening outcome was examined in relation to age, race, ethnicity, primary language, and trimester of the prenatal screen. Crude and adjusted odds ratios were generated from logistic regression models.

***Results:*** Overall, 16.4% of women screened positive for depression. English-speaking women, non-Hispanic white women, and those ages >35 years were more likely to screen positive. Women >35 years also had higher odds of reporting thoughts of self-harm than younger women.

***Conclusion:*** In this low-income border population, non-Hispanic white, English-speaking women over the age of 35 were at the greatest risk of peripartum depression. These findings underscore the need for peripartum depression screening in this population.

## Introduction

Population-based data show that 12.8% of reproductive-age women experience depression, and one or more chronic disease risk factors, which may impact their well-being and future pregnancies.^1^ Depressed women, as compared with nondepressed counterparts, report a higher prevalence of cardiovascular disease, diabetes, smoking, substance use disorder, obesity, and physical inactivity. Depression is one of the most common complications in pregnancy and has been associated with adverse birth outcomes.^2^

Peripartum depression is a diagnosis of major depressive disorder occurring during pregnancy or within a short time after delivery. The Diagnostic and Statistical Manual of Mental Disorders–V uses the specifier of peripartum onset and defines it as the most recent episode occurring during pregnancy (prenatal depression) or within 4 weeks of delivering a child (postpartum depression).^3^ The current recommendation is to consider not only depressive symptoms that begin after delivery but also the symptoms that emerge during pregnancy.^4,5^

### Peripartum depression: consequences for mother and child

Multiple consequences may develop from untreated peripartum depression, including maternal, fetal, infant, and child effects. Depressed women report reduced sleep, poor appetite, and weight gain, less frequent prenatal care visits, and self-medication with tobacco, alcohol, drugs, and suicide during pregnancy and postpartum.^6^ Additional maternal risks associated with peripartum depression include spontaneous abortion, low birth weight, bleeding during gestation, cesarean delivery, and preeclampsia. In extreme cases, untreated depression may lead to suicide during pregnancy and postpartum. Moreover, untreated peripartum depression yields long-term consequences. Farr et al.^1^ report that women with a history of depression experience elevated odds of having three chronic conditions and risk factors, as compared with women with no depression. Moreover, reproductive-age women with current depression had 1.5 to 3.7 times the odds of having one or more chronic conditions and risk factors, as compared with nondepressed counterparts.

Previous studies show that peripartum depression endangers young children's cognitive, socioemotional, and behavioral development, as well as their learning and physical and mental health over the long term. For example, Kozhimannil^7^ reported a higher frequency of depressive avoidant attachment, reduced cognitive/emotional/verbal and social skills up to puberty. Adolescents exposed to mothers with episodic postpartum depression reported significantly lower intelligence quotient among boys and psychological disorders in girls.^8^ Similarly, Pawlby et al.^9^ found that the risk of depression for the 16-year-olds exposed to antenatal depression was 4.7 times greater than for counterparts who were not exposed to peripartum depression.

Rates of peripartum depression are higher among underserved mothers of low socioeconomic status and racial/ethnic minorities as compared with the general population.^2^ In the general population, however, depressive symptoms affect more than 25% of peripartum women, with approximately 10%–15% of mothers experiencing a major depressive disorder.^10,11^

The New Mexico Pregnancy Risk Assessment Monitoring System (PRAMS) found that 20% of New Mexico mothers in 2004 reported postpartum depressive symptoms, which was the highest rate among 17 PRAMS states.^12^ The rate of depression symptoms in the other PRAMS populations varied from 11% to 20%.

From 1999 to 2019, the Doña Ana Healthy Start (DAHS) program provided prenatal support and education, child development guidance, and local community resource information to low-income families with infants, toddlers, and pregnant women in southern New Mexico. DAHS recruited pregnant women into early prenatal care and parenting families into interconceptional care and infant case management services. Caseworkers conducted home visitations to provide targeted case management retention interventions. At the onset, case management services consisted of biopsychosocial assessments and individualized care plans, in addition to peripartum and interconception visits. However, in 2001, all sites in the Healthy Start program added maternal depression screening and referral to their services.^13^ Although some Healthy Start programs documented and published their research,^9^ little quantitative research on maternal depression has been systematically conducted in predominantly Hispanic Healthy Start populations, such as that of southern New Mexico.

Further research on this population is vital given the intersection of socioeconomic, cultural, political, and linguistic features that define the borderland. This multifaceted reality represents social determinants of mental health. Indeed, disproportionate poverty, high unemployment, uninsured rates, insecure residency status, and limited transportation restrict access to quality health care services for a structurally vulnerable and marginalized immigrant borderland population.^14–17^ Limited information about health risks in this population hampers capacity to address local needs and assess the effectiveness of public health programs.^18,19^ To address this gap, we studied Healthy Start participants who received peripartum services, including depression screening, in southern New Mexico. This large community sample provides an excellent opportunity to obtain a more reliable measure of peripartum depression in a particularly vulnerable, borderland population.

We captured depression throughout the pregnancy and postpartum periods. We aimed to: (1) describe the demographic characteristics of the DAHS population, (2) examine the prevalence of clinical symptoms of depression during the peripartum period, and (3) explore the relationships between positive screening scores and demographic characteristics.

## Materials and Methods

### Study design

The study is a secondary data analysis of case series observational data collected as part of regular peripartum services provided to women in southern New Mexico. Participants of the Healthy Start program received outreach, case management, health education, depression screening, care coordination, and interconceptional care services. Specific program services, as well as national evaluation reviews of Healthy Start programs, are provided elsewhere.^20^ The Institutional Review Board of the authors' institution approved this study protocol.

### Participants

A criterion sample was obtained from women receiving services between August 2009 and January 2017. According to the DAHS postpartum period definition, eligibility was restricted to pregnant women in their first to third trimester and up to 8 weeks postpartum. Women who received services during the interconceptional period only were excluded from the study.

#### Screening outcomes measurement and definitions

Peripartum depression symptoms were measured by the Edinburgh Postnatal Depression Scale (EPDS). The 10-item EPDS is the most commonly used peripartum screening tool for depression. It has been shown to be valid and reliable and has been normed on English and Spanish-speaking mothers.^21^

The EPDS was orally administered in English or Spanish by DAHS caseworkers as preferred by the DAHS clients. EPDS items are scored on a 4-point scale (0 to 3). The total depression score for each screen is obtained by summing the scores for each for the 10 EPDS questions.^22^ Various cutoffs have been employed as an indication of a positive screening.^23^ For this study, a cutoff of 10 was used to indicate a positive screening as this level has been found to have a sensitivity that ranged from 78% to 100% and specificity that ranged from 44% to 89%.^24^ In addition, suicidal ideation was measured with a positive response to question 10, “the thought of harming myself has occurred to me.”

The data were analyzed according to the period during which the EPDS screening(s) took place. Data structure did not allow for longitudinal analyses of depression. A woman was considered to have screened positive for depression during the prenatal period if her initial prenatal screen was positive, during the postpartum period if her initial postpartum screen was positive, and during the peripartum period if she had at least one positive screen during the prenatal or postpartum period. Total peripartum screening outcomes were determined based on all prenatal and postpartum screens for a given woman.

### Demographic characteristics

In addition to depression screening results, the data included the mother's date of birth, race, ethnicity, primary language, date of service, trimester of pregnancy (for prenatally screened women), and father's employment and family size. Screenings per woman varied depending on when the woman entered the program, her length of time in the program, and the results of any prior screens. Age was calculated as the difference between a woman's date of birth and the date of service. To compare our results with other literature,^25^ age was grouped as <20, 20–35, or >35 years. Race was categorized as white or non-white; ethnicity as Hispanic or non-Hispanic; and primary language as English or non-English. Interview language (English vs. Spanish) was used as an indicator of acculturation.

### Data analysis

The percentages of women with an EPDS screening score of 10 or above were computed for each demographic variable for the prenatal, postpartum, and peripartum periods. The 95% confidence intervals (CIs) for percentages were computed using the Clopper–Pearson method.^26^ Chi-square tests of independence and, when expected counts were small, Fisher's exact tests were performed to explore the statistical significance of associations between each demographic variable and a positive screen.

Associations between each demographic variable and a positive screen were quantified by unadjusted and adjusted estimated odds ratios (ORs) generated from logistic regression models. For the prenatal and postpartum periods, demographic variables were measured at a woman's initial screen during the period. For the peripartum period, demographic variables were measured at a woman's first screen during the prenatal or postpartum period. For the prenatal period, trimester (first, second, or third) was included in the adjusted model. Women with missing values for a demographic variable were omitted from models that included that variable. All data processing and analyses were performed using SAS version 9.4. A significance level of 0.05 was used.

## Results

### Demographic and screening characteristics

Between 2009 and 2017, a total of 1453 women in the DAHS program received at least one EDPS screening during the prenatal or postpartum period (Table 1). Women ranged in age from 13 to 46 years (mean = 25.0, standard deviation = 6.4). Over 70.0% of women were between the ages of 20 and 35. The majority of women (97.6%) reported their race as white, 93.0% identified their ethnicity as Hispanic, and 62.9% spoke Spanish as their primary language. Most women were initially screened during the first or second trimester. The number of screenings per woman ranged from 1 to 9, with 48.3% of women screened once, and 29.5% screened twice. Of 2673 total screenings, 22.7% took place during the first trimester, 23.8% during the second trimester, 27.7% during the third trimester, and 25.8% postpartum (data not shown).

**Table 1.** Characteristics of 1453 Women in the Doña Ana Healthy Start Program, 2009–2017

	Frequency^a^	%
Age at 1st screening (years)		
<20	317	21.9
20–35	1025	70.8
>35	105	7.3
Race^b^		
White	1403	97.6
Non-white	34	2.4
Ethnicity^c^		
Hispanic	1336	93.0
Non-Hispanic	101	7.0
Primary language		
English	506	35.8
Spanish	890	62.9
Other	18	1.3
Period at 1st screening		
First trimester	537	37.0
Second trimester	576	39.6
Third trimester	284	19.6
Postpartum	56	3.9
Number of screenings		
1	701	48.3
2	428	29.5
3	241	16.6
4	43	3.0
5 or more	40	2.8

^a^ Numbers do not sum to 1453 due to missing values.

^b^ Black, Indian, Chinese, Filipino, other Asian, and other race were combined as non-white.

^c^ Mexican, Spanish, Central or South American, and Puerto Rican were combined as Hispanic while non-Spanish white (Anglo), not Hispanic or Latino, and Native American were combined as non-Hispanic.

### Prevalence of depression

Table 2 presents the frequencies and percentages of women with a positive depression screen (EPDS score 10 or above) during the prenatal, postpartum, and peripartum periods between 2009 and 2017.

**Table 2.** Prevalence of a Positive Screening Score by Demographic Characteristics During the Prenatal (*n* = 1401), Postpartum (*n* = 611), and Peripartum (*n* = 1453) Periods, 2009–2017

	Initial prenatal screening			Initial postpartum screening			All peripartum screenings		
	*n*	% (95% CI)	*p*^a^	*n*	% (95% CI)	*p*^a^	*n*	% (95% CI)	*p*^a^
Overall	178	12.7 (11.0–14.6)		58	9.5 (7.3–12.1)		238	16.4 (14.5–18.4)	
Age			0.46			**0.02**			**0.02**
<20	40	13.1 (9.5–17.4)		14	12.6 (7.1–20.3)		55	17.4 (13.4–22.0)	
20–35	122	12.3 (10.3–14.5)		32	7.4 (5.1–10.3)		155	15.1 (13.0–17.5)	
>35	16	16.7 (9.8–25.7)		11	16.7 (8.6–27.9)		27	25.7 (17.7–35.2)	
Race			0.59			1			0.82
White	169	12.5 (10.7–14.3)		56	9.5 (7.2–12.1)		227	16.2 (14.3–18.2)	
Non-White	5	15.6 (5.3–32.8)		1	6.7 (0.2–32.0)		6	17.7 (6.8–34.5)	
Ethnicity			0.12			**0.02**			**0.03**
Hispanic	157	12.2 (10.4–14.1)		49	8.6 (6.5–11.3)		209	15.6 (13.7–17.7)	
Non-Hispanic	17	17.5 (10.6–26.6)		8	20.5 (9.3–36.5)		24	23.8 (15.9–33.3)	
Primary language			0.09			0.63			**0.04**
Spanish	96	11.2 (9.1–13.4)		33	8.5 (5.9–11.7)		128	14.4 (12.1–16.9)	
English	75	15.3 (12.2–18.8)		21	10.8 (6.8–16.0)		99	19.6 (16.2–23.3)	
Other	2	11.8 (1.5–36.4)		1	12.5 (0.3–52.7)		3	16.7 (3.6–41.4)	
Trimester			0.37						
First	63	11.7 (9.1–14.7)							
Second	72	12.5 (9.9–15.5)							
Third	43	15.1 (11.1–19.8)							

EPDS score 10 or above. Bolded text represents statistically significant findings.

^a^ Chi-square tests of independence and, when expected counts were small, Fisher's exact tests were performed.

CI, confidence interval; EPDS, Edinburgh Postnatal Depression Scale.

#### Prenatal period

Of the 1401 women who were screened at least once during the prenatal period, 178 (12.7%) scored 10 or above in their initial prenatal screening. At the initial prenatal screening, English-speaking women were more likely than Spanish-speaking women to report a score of 10 or above (15.3% vs. 11.2%).

#### Postpartum period

Among the 611 women screened at least once during the postpartum period, 58 (9.5%) scored 10 or above at their initial postpartum screening. During this period, women 35 years or older (16.7%) were significantly more likely to report a score of 10 or above, as compared with younger counterparts (*p* = 0.02). Further analysis (not shown) found 11 of 611 (1.8%) women reported thoughts of harming themselves at the initial postpartum screening. Moreover, women who identified as Hispanic were significantly less likely to report a score of 10 or above (8.6%) than non-Hispanic women (20.5%, *p* = 0.02).

#### Peripartum period

Of the 1453 women with at least one peripartum depression screening, 238 (16.4%) had at least one score greater than or equal to 10, and 295 (20.3%) had at least one score greater than or equal to 9 (data not shown). Women 35 years or older (25.7%) were significantly more likely to report a score of 10 or above, as compared with younger counterparts (*p* = 0.02). In addition, for all peripartum screenings, Hispanic women were significantly less likely to report a score of 10 or above, as compared with non-Hispanic women (*p* = 0.03). Finally, for all peripartum screenings, women whose primary language was Spanish (14.4%) were significantly less likely to report a score of 10 or above, as compared with English or other primary language speakers (*p* = 0.04).

### Risk factors for positive screening scores

Table 3 presents the unadjusted and adjusted ORs for a positive depression screen based on initial prenatal, initial postpartum, and all peripartum screenings.

**Table 3.** Estimated Odds Ratios and 95% Confidence Intervals for a Positive Depression Screening by Demographic Characteristics During the Prenatal (*n* = 1401), Postpartum (*n* = 611), and Peripartum (*n* = 1453) Periods, 2009–2017

	Initial prenatal screening		Initial postpartum screening		All peripartum screenings	
		Adjusted^a^		Adjusted^b^		Adjusted^b^
	Unadjusted	*n* = 1348	Unadjusted	*n* = 591	Unadjusted	*n* = 1393
Age						
>35 vs. ≤35 years	1.40 (0.80–2.46)	1.43 (0.78–2.62)	2.17 (1.06–4.42)^*^	2.44 (1.17–5.12)^*^	1.87 (1.18–2.96)^**^	2.04 (1.26–3.32)^**^
Race						
Non-white vs. white	1.30 (0.50–3.43)	1.11 (0.38–3.26)	0.68 (0.09–5.29)	0.35 (0.04–3.19)	1.11 (0.45–2.71)	0.89 (0.33–2.39)
Ethnicity						
Non-Hispanic vs. Hispanic	1.53 (0.89–2.66)	1.27 (0.67–2.40)	2.73 (1.19–6.26)^*^	2.79 (1.02–7.68)^*^	1.68 (1.04–2.72)^*^	1.40 (0.80–2.45)
Primary language						
English vs. non-English	1.44 (1.04–1.98)^*^	1.46 (1.03–2.07)^*^	1.29 (0.73–2.29)	1.20 (0.63–2.27)	1.44 (1.08–1.92)^*^	1.48 (1.09–2.02)^*^
Trimester						
Second vs. first	1.08 (0.75–1.55)	1.01 (0.69–1.47)				
Third vs. first	1.34 (0.88–2.04)	1.33 (0.86–2.04)				

EPDS score 10 or above. Bolded text represents statistically significant findings.

^**^ *p* < 0.01.

^*^ *p* < 0.05.

^a^ Adjusted for age, race, ethnicity, primary language, and trimester.

^b^ Adjusted for age, race, ethnicity, and primary language.

#### Prenatal period

At the initial prenatal screening, English-speaking women were more likely to screen positive for depression as compared with non-English-speaking women (adjusted OR = 1.46, 95% CI = 1.03–2.07).

#### Postpartum period

At the initial postpartum screening, women older than 35 years had significantly higher odds of scoring 10 or above on the EPDS than women 35 years old or younger (adjusted OR = 2.44, 95% CI = 1.17–5.12). Non-Hispanic women had significantly higher odds of scoring 10 or above than Hispanic women (adjusted OR = 2.79, 95% CI = 1.02–7.68).

#### Peripartum period

For all peripartum screening, women older than 35 years had a significantly higher odds of scoring 10 or above (adjusted OR = 2.04, 95% CI = 1.26–3.32), as compared with their younger counterparts. In the unadjusted OR, non-Hispanic women had a significantly higher odds of scoring 10 or above (OR = 1.68, 95% CI = 1.04–2.72). English-speaking women reported a higher risk for depression as compared with their non-English-speaking counterparts (adjusted OR = 1.48, 95% CI = 1.09–2.02). For a subset of women, the mother's and father's insurance, education, employment, estimated family income, and family size were available. Among this subset, women with a family size of 2 were significantly more likely to be depressed as compared with women with a family size greater than 2 (adjusted OR = 3.11, 95% CI = 1.19–8.13, data not shown).

#### Positive thoughts of self-harm

Additional analysis (not shown) was conducted for question 10 (positive for thoughts of self-harm). At the initial prenatal screening, 5.2% of women older than 35 years (5 of 96) scored positive for thoughts of self-harm versus 1.6% of women 35 years old or younger (21 of 1299). Older women had significantly higher odds of scoring positive for thoughts of self-harm than younger women (adjusted OR = 4.45, 95% CI = 1.56–12.69). This elevated risk for women older than 35 years was also found for all peripartum screenings, with 6.7% of women older than 35 years (7 of 105) scoring positive for thoughts of self-harm versus 2.5% of women 35 years old or younger (33 of 1342, adjusted OR = 3.38, 95% CI = 1.41–8.08).

## Discussion

This study examined the prevalence of clinical symptoms of depression during the peripartum period among women seeking Healthy Start maternal and child health services in one southern New Mexico county. Among the 1453 prenatal and postpartum women screened for depression in the DAHS program, 12.7% were at high risk of depression (EPDS score 10 or above) during their prenatal period and 9.5% during the postpartum period. Women older than 35 years of age were significantly more likely to report thoughts of harming themselves, as compared with their younger counterparts. This finding is consistent with other studies that found evidence of increased suicidal ideation among perinatal women.^27^

Also consistent with previous findings, the percentage of all study women having an EPDS screening score of 10 or above at least once during the peripartum period was 16.4%, and 9 or above was 20.3%. This prevalence rate is on par with peripartum depression prevalence rates in New Mexico,^28^ elsewhere in the U.S.–Mexico border region, and other Healthy Start programs serving minority rural communities^29^ that used various instruments to screen for depression. This finding was also consistent with the result from a 2010 pilot project conducted by the Families FIRST Case Management Program,^12^ a New Mexico program that worked closely with the DAHS to provide peripartum obstetric services to women.

Several investigators used EPDS to examine prenatal and postpartum depression in different Healthy Start programs. Consistent with the prevalence of depression in our study, an examination of the Omaha Healthy Start (*n* = 119 women) found that 16.0% of women screened positive for depression.^23^ Of the 1800 women screened in the Des Moines Healthy Start Project, 32.3% scored positive for depression using a cutoff of 12 or above.^13^ The higher prevalence compared with our study may be due, in part, to the lower percentage of Hispanic women in the Des Moines Healthy Start population (45%). Similarly, the Tampa Healthy Start Coalition (*n* = 180 women) found that 35% of women screened positive for depression, using a cutoff of 11 or above.^30^ This percentage, which was based on a small sample size (*n* = 180), was higher than in the current study and may be due to differences in key demographic sample characteristics like percentages of single and first-time mothers.

### Factors that may have an impact on the risk of depression

In our study, significantly higher percentages of depressive symptoms and self-harm ideation were detected for women older than 35 compared with younger women during the peripartum period. Similar results were found in the Canadian Community Health Survey study that analyzed depression data between 2007 and 2008.^31^ This finding of self-harm ideation during the peripartum highlights the need for screening and intervention. The Confidential Enquiry into Maternal Deaths study found that suicide was the leading cause of maternal death in the first postpartum year.^6^

However, concerning age, our results were different from New Mexico PRAMS results, which showed that the percentage of mothers who reported postpartum depressive symptoms decreased with increasing age.^12^ Our study found that postpartum women younger than age 20 had a higher risk of depression compared with postpartum women ages 20 to 35. Still, women older than 35 also had a higher risk compared with women ages 20 to 35. The difference in findings may be due to our study's small number of women over the age of 35. In addition, PRAMS results are based on a different, two-question measure of postpartum depressive symptoms.^12^

In our study, language and ethnicity served as indicators for acculturation.^32,33^ Language and ethnicity were significantly related to depression, suggesting that childbearing Hispanic women with higher acculturation may be at higher risk of adverse mental health outcomes than similar women with lower acculturation.^23^ Such findings are consistent with the Hispanic epidemiological paradox.^34^ As an explanatory model, this concept posits that among socially and economically disenfranchised immigrants, traditional culture influences health behaviors, which translates into favorable health outcomes.^27^ In our study, the odds of an EPDS score of 10 or above for Spanish speakers were 32% less than for English speakers. In addition, we found that Hispanic women were less likely to be depressed than non-Hispanic women, lending further support to the Hispanic epidemiological paradox. In initial postpartum screenings, the percentages of Hispanic women with a high EPDS score were significantly lower than those for non-Hispanic women. Across all peripartum screenings, the odds of having a score of 10 or above were 40% higher for non-Hispanic women than for Hispanic women. The pattern of these findings supports the literature that acculturation among Hispanic women is associated with increased risk. Evidence suggests that acculturative stress negatively impacts maternal depressive symptoms.^35^ Indeed, perinatal depression coupled with the socioeconomic and immigration stressors gradually elevate the risk for comorbid disorders, such as posttraumatic stress disorder, generalized anxiety disorder, and bipolar depression.^6^ Community surveillance data show that in 2017, the New Mexico prevalence of mental distress (6+ days) was 20.2%, significantly higher compared with that of the United States (18.4%).^36^ For some women, untreated depression can have tragic comorbid consequences that lead to increased chronic disease risk factors, like obesity and diabetes, and the most serious being death by suicide. To the extent that depression may contribute to these chronic illnesses, secondary prevention efforts related to peripartum depression could have multiple beneficial effects.

### Implications for practice and policy

Our findings highlight the heterogeneity of the prevalence of depression and varied risk factors that affect women. These findings emphasize the necessity of peripartum depression screening and the importance of establishing protocols for timely and consistent evaluation of depression throughout pregnancy and the postpartum period.^37^ The significant relationships between ethnicity, language, age, and depression rates found in this study provide essential indicators that inform further practice and research. The socioeconomic status, immigration status, anti-immigrant biases, poor spousal relationships, and cultural reality of borderland women produce a nexus of social determinants of health factors that underscores the critical importance of promoting an intersection of public health and health care.^38^ As such, efficacious treatment modalities for peripartum depression encompass conceptual frameworks that recognize the importance of culturally specific care for mothers and respect the diverse cultural beliefs about child-rearing and mothering.^14^ For example, programs that incorporate promotoras (community health workers) to strategically screen and address maternal depression are more effective than the standard services offered at most primary care clinics serving pregnant women.^39–41^ Additionally, best-practice models have shown that effective care of maternal depression requires a systematic approach to overcome the sociocultural barriers to self-disclosure.^23^

Moreover, the integration of mental health services with prenatal and postpartum care, with a patient-centered approach, is more effective than a traditional medical model of care in the engagement of at-risk women during their pregnancy.^29,42^ Integrated behavioral health models that include rounds with social workers, psychiatry, psychology, obstetrics and gynaecology, family, or pediatric physicians and nurses provide a foundation to address the complex needs of at-risk women. In fact, compliance with recommended care is significantly higher for women offered integrated onsite care.^21,30^ In future research, a more in-depth study of factors associated with specific subgroups of women with elevated risk is fundamental in the consideration of best-practice for screening and treatment of peripartum depression. Additional research is needed to further our understanding of borderland women's peripartum health, the barriers they experience, and their treatment preferences. The detection and treatment of poor mental health may moderate lifelong chronic conditions and risk factors known to be prevalent in this population.^1^ Such efforts can lead to a more nuanced and culturally specific identification, referral, and intervention, as well as improved long-term health.

### Limitations

Our findings should be interpreted in light of study limitations. The majority of participants in this study's population were white (98%) and Hispanic (93%) women attending a peripartum care program in one southern New Mexico County. This homogeneity in the study population made it harder to compare the differences in risk of depression among race and ethnicity due to the very small sample sizes in the non-white and non-Hispanic groups. An additional limitation is that the prevalence of depression in the DAHS study population may be underestimated. Border culture and social norms, coupled with substantial socioeconomic barriers to help-seeking behaviors, may prevent women from seeking attention.^43^ Moreover, some clinicians express concern that women may interpret questions differently depending on their cultural background.^19^ However, the EPDS is considered a sensitive and valid screening tool for peripartum depression for refugee and Spanish-speaking women.^21^

## Conclusions

Between 2009 and 2017, 1453 women in a Healthy Start program received at least one EDPS screening during the prenatal or postpartum period. In our study, of the 1453 women with at least one peripartum depression screening, 16.4% had at least one score greater than or equal to 10, and 20.3% had at least one score greater than or equal to 9. Women older than 35 years had significantly higher odds of scoring 10 or above, as compared with their younger counterparts. Finally, women older than 35 years had significantly higher odds of scoring positive for thoughts of self-harm than younger women. These findings underscore the need for peripartum depression screening in this population.

## Abbreviations Used

CI: confidence interval
DAHS: Doña Ana Healthy Start
EPDS: Edinburgh Postnatal Depression Scale
OR: odds ratio
PRAMS: Pregnancy Risk Assessment Monitoring System
